# Role of T Cell Receptor Affinity in the Efficacy and Specificity of Adoptive T Cell Therapies

**DOI:** 10.3389/fimmu.2013.00244

**Published:** 2013-08-21

**Authors:** Jennifer D. Stone, David M. Kranz

**Affiliations:** ^1^Department of Biochemistry, University of Illinois, Urbana, IL, USA

**Keywords:** adoptive T cell therapy, TCR affinity, T cell sensitivity, T cell cross-reactivity, tumor-associated epitopes

## Abstract

Over the last several years, there has been considerable progress in the treatment of cancer using gene modified adoptive T cell therapies. Two approaches have been used, one involving the introduction of a conventional αβ T cell receptor (TCR) against a pepMHC cancer antigen, and the second involving introduction of a chimeric antigen receptor (CAR) consisting of a single-chain antibody as an Fv fragment linked to transmembrane and signaling domains. In this review, we focus on one aspect of TCR-mediated adoptive T cell therapies, the impact of the affinity of the αβ TCR for the pepMHC cancer antigen on both efficacy and specificity. We discuss the advantages of higher-affinity TCRs in mediating potent activity of CD4 T cells. This is balanced with the potential disadvantage of higher-affinity TCRs in mediating greater self-reactivity against a wider range of structurally similar antigenic peptides, especially in synergy with the CD8 co-receptor. Both TCR affinity and target selection will influence potential safety issues. We suggest pre-clinical strategies that might be used to examine each TCR for possible on-target and off-target side effects due to self-reactivities, and to adjust TCR affinities accordingly.

## Introduction

Immunotherapies of cancer use either passive or active approaches to recruit immune cells against tumor cells. Although most passive strategies to date have involved monoclonal antibodies, a growing body of work shows that T cells may provide more immediate and potent anti-tumor cell activity. In the most common adoptive T cell approaches under investigation, genes that encode a T cell receptor (TCR) or a chimeric antibody-based receptor (chimeric antigen receptor, CAR) are introduced into *ex vivo* activated T cells from a patient. Both receptors have shown significant promise, but the properties of these receptors that yield the most effective responses continue to be explored. In addition, because of their potency and sensitivity, adoptive T cells can present safety issues that have not generally been seen with antibodies. Aspects of TCR-mediated adoptive T cell approaches are reviewed here.

## TCR-Mediated Adoptive T Cell Therapies

It has been a reasonable tenet that the potency of TCR-mediated adoptive T cell therapies could be improved by using class I-restricted TCRs that are able to function both in their normal context, CD8 T cells, and in CD4 T cells. While CD8 T cell activities against cancer are important, recruitment of CD4 T cells to the site of a tumor can result in direct tumor control (1) and provide a cytokine milieu that promotes the function and survival of CTLs and NK cells (2–9), and CTL proliferation within tumors (10). CD4 T cells can also take on a cytotoxic phenotype, killing tumor cells directly (11, 12). Finally, CD4 T cells contribute to IFNγ-dependent mechanisms of angiogenesis inhibition (13, 14) and enhanced innate and adaptive responses (15, 16).

The recruitment of CD4 T cells with class I MHC-restricted TCRs is, however, confounded by the fact that most TCRs with class I specificity require co-expression of CD8 for full activity. Nevertheless, some TCRs have been shown to mediate activity without CD8 suggesting that they have higher “functional avidity” (7, 17–23). Experimental studies using CD8 binding-impaired MHCs (24) or T cells that do or do not express co-receptor (25, 26) have defined affinity thresholds above which TCRs can respond to class I MHC without a requirement for CD8. There are now many approaches available to isolate or engineer TCRs that exhibit higher affinities and thus, act independent of CD8 (27–32).

## Role of CD8 in Enhancing T Cell Sensitivity

The dual roles of the CD8 co-receptor in binding to the class I MHC ligand and in signaling have been the topic of many investigations. The synergy between the TCR and CD8 allows just a few class I complexes on a target cell to stimulate cytolysis (33, 34). This exquisite sensitivity has evolved to allow our immune system to identify a potential target cell as “foreign” under conditions where the processed antigen levels are extremely low.

It has been argued that CD8 functions primarily by bringing the intracellular kinase Lck together with the TCR/CD3 complex (35). It should also be noted that CD8 binding to non-cognate pepMHC has a profound impact on increasing T cell sensitivity, and that the overall surface density of pepMHC is important in the contribution of CD8 (36, 37). Accordingly, MHC density on tumor cells can play a role in the function of both CD8 and the antigen-specific TCR.

Regardless of the exact mechanism, CD8 synergy with the TCR is so effective that cytolytic activity of CTLs can be induced even with very low TCR affinities [e.g., 300 μM (38, 39)]. This might be particularly important in the case of CD8 T cell responses against self-cancer antigens, where the TCR affinities appear to be lower than TCR affinities against foreign antigens (40, 41), most likely due to negative selection in the thymus. The TCR affinity threshold in the thymus that promotes negative selection is thought to be set very low in order to reduce the risk of peripheral autoimmune reactions (42–46). However, the well-known ability of CD8 to synergize with very low affinity TCRs also presents issues of undesirable autoreactivities against structurally similar self-peptides, when the affinity of the TCR against the cognate tumor antigen is increased (see below).

## TCR:pepMHC Affinities

Given the central role of TCR affinity in both driving T cell activity and in conferring the specificity of the reaction, we summarize concepts of affinity and its measurement here. More thorough reviews have been published elsewhere [e.g., (40, 47, 48)]. One straightforward way to describe TCR binding to pepMHC is as a simple, one-to-one interaction involving a bimolecular binding reaction:

$$\text{TCR}+\text{pepMHC}\overset{k_{\text{on}}}{\underset{k_{\text{off}}}{⇌}}\text{TCR:pepMHC}$$

where *k*_on_ indicates the association rate of the interaction, and *k*_off_ describes the dissociation rate of the interaction. Additional parameters describing the binding can be determined from these association and dissociation rates, including the half-life [*t*_1/2_ = ln(2)/*k*_off_] and the equilibrium binding constant (*K*_d_ = 1/*K*_a_ = [TCR][pepMHC]/[TCR:pepMHC] = *k*_off_/*k*_on_). The equilibrium binding constant may also be measured with equilibrium (or estimated from quasi-equilibrium) binding experiments, using techniques such as Scatchard plots or other fitting of the bound vs. free equation for *K*_d_. In this review, we do not describe the key role of peptide affinity for the MHC product, but this parameter is also critical in the assessment of which peptide(s) to target (49–52).

The bimolecular binding equation above is used to describe the interaction between two free molecules in solution, with 3D mobility. Using soluble versions of pepMHC and/or TCR and measurement techniques such as binding to cell surfaces or surface plasmon resonance, a variety of models relating TCR binding parameters to T cell triggering have been developed (40). These included models based on the dissociation rate (*k*_d_) such as “kinetic proofreading” (53), which suggested that a critical *t*_1/2_ threshold must be exceeded for T cell activation to occur. An extension of this model proposed an “optimal dwell time” (54), incorporating the concept that exceptionally long *t*_1/2_ values would result in reduced activity at low antigen density as a consequence of reduced serial triggering of multiple TCRs by each cognate pepMHC molecule (55). This model, which predicts reduced sensitivity of TCRs with long half-lives seems to be contradicted by very high-affinity TCRs engineered via directed evolution that can mediate sensitive T cell responses to low amounts of antigen (56).

Because the TCR, CD8, and pepMHC all exist as integral cell surface proteins on opposing cells, each present in various numbers, the corresponding multivalent interactions have been difficult to deconvolute from cell-free affinity measurements. Initial exceptions to the correlation between *k*_off_ and activity among TCR:pepMHC pairs led to consideration of the value of *k*_on_ in the overall interaction (57–59). In the 2C system, which benefits from a large repertoire of reagents, measurements of pepMHC affinities by competition with a TCR clonotypic antibody on the live T cell surface gave good correlation with sensitivity and activity of 2C T cells against those targets (38). This approach allows direct measurement of the cell surface affinities, but unfortunately due to the lack of appropriate antibody reagents, most TCRs can not be probed in this manner. More recently, using careful statistical analyses and experimentation, a confinement time model of TCR triggering highlighted the contribution of *k*_on_ and potential re-binding of the same TCR:pepMHC (60). *In situ* measurements of TCR:pepMHC binding to opposing 2D surfaces were also performed, using single-molecule fluorescence resonance energy transfer (61) or mechanical force and contact surface area measurements (62). These studies revealed that binding parameters were altered/accelerated under the more physiological geometries, showing high correlation between faster on-rates, lower 2D-*K*_d_ values, and more potent agonist activity.

Regardless of the type of *K*_d_ measurement, 2D or 3D, or the involvement of kinetics, it is reasonable to conclude that TCR:pepMHC systems exhibit a: (1) minimum affinity threshold required to be stimulated by cognate pepMHC, (2) a maximum affinity threshold above which there is no longer improvement in sensitivity (or even a reduction in sensitivity), and (3) that these affinity-minima and -maxima will have different ranges, depending on whether the cognate co-receptors (CD8 for a class I pepMHC and CD4 for a class II pepMHC) are present.

## Role of TCR Affinity in Mediating Activity of CD4 and CD8 T Cells Against a Class I MHC Antigen

**Figure 1:**
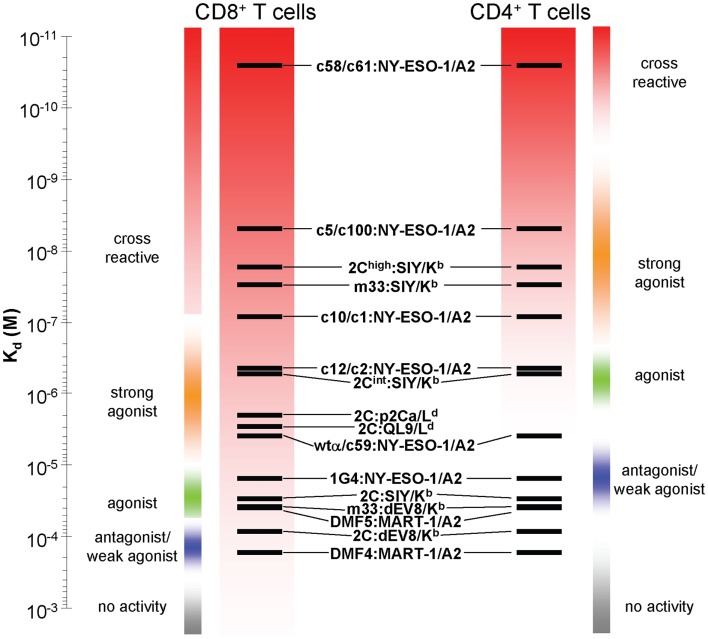
**Relationship between T cell activities and TCR affinities for a class I pepMHC antigen, in either CD8 or CD4 T cells**. Various TCRs whose affinity for their target class I pepMHC complexes have been measured are depicted on an affinity scale (*K*_d_). The relative activity ranges for those receptors are listed for those TCRs expressed in CD8 (left) and CD4 (right) T cells. The activity boundaries are approximated from the best-known systems. Sensitivity at low TCR affinities is achieved due to TCR synergy with the CD8 co-receptor. This same principle can yield CD8-dependent, undesirable cross reactivities with structurally similar self-peptides.

Class I MHC is engaged by the CD8 co-receptor with relatively low affinity (*K*_d_ ∼ 10–200 μM), that varies by allele (35, 63–67). Nevertheless, CD8 participation can increase sensitivity of a T cell to its cognate class I pepMHC complex by one-million fold (56), reviewed in (67). Accordingly, in the targeting of class I pepMHC, normal wild-type affinity TCRs in the range of 10–300 μM [reviewed in (40)] are sufficient to provide very sensitive responses (Figure 1). Indeed, normal CD8 T cells have been shown to respond to as few as one to three agonist pepMHC complexes on the surface of a cell (33, 34) due to the synergy with CD8. The ability of CD8 to synergize with even very low affinity TCRs [*K*_d_ > 300 μM (25, 67, 68)] can be advantageous in the normal anti-tumor setting, as most anti-self (and, hence, anti-tumor) pepMHC reactive T cells would have been deleted in the thymus if they exhibited even modest affinities. Based on studies with various TCRs against class I pepMHC, the minimal affinity required for CD8 T cell activity appears to be in the range of 300 μM, whereas the optimal affinity above which there is no additional *in vitro* or *in vivo* improvement is about 10 μM (24, 26, 69, 70). However, there has been some evidence that higher-affinity TCRs yield faster T cell reactions, but reduced sensitivity at lower pepMHC densities (71, 72).

As indicated, it has been shown that CD4 T cell responses against tumors are very beneficial, a process that can be achieved by transducing CD4 T cells with TCRs that have higher affinities (*K*_d_ < 10 μM) against a class I MHC tumor antigen (25, 29, 73) (Figure 1). Even for CD4 T cells, however, there seems to exist an affinity threshold for class I pepMHC above which T cell activation occurs in the absence of the cognate peptide, as was seen for a picomolar-affinity TCR against HLA-A2/NY-ESO-1 (157–165) (73). This CD4 T cell activation appears to be due to the interaction of the affinity-engineered TCR with one or more self-pepMHC complexes with affinities above the CD8-independent threshold (i.e., *K*_d_ < 10 μM).

Raising the affinity of a TCR in order to achieve optimal CD4 T cell activity (i.e., CD8 independence) also increases the risk that the same TCR, in a CD8 T cell, will mediate activity against structurally related self-peptides. In this scenario, TCR affinities for such a self-peptide-MHC that were below the threshold (e.g., *K*_d_ > 300 μM, in the presence of CD8) for the wild-type TCR may now be elevated to<300 μM with the affinity-enhanced TCR. In summary, in CD4 T cells a high-affinity TCR against a cognate pepMHC would need to cross-react with a structurally related self pepMHC at an affinity of at least 10 μM to stimulate autoreactivity, whereas in CD8 T cells a high-affinity TCR against a cognate pepMHC would need to cross-react with a structurally related self pepMHC at an affinity of only 300 μM to stimulate autoreactivity, due to the synergy of CD8.

The consequences of these self-peptide cross-reactions can be varied. In one case (see 2C system below), a higher-affinity TCR introduced into CD8 T cells resulted in self-peptide reactivity and rapid deletion of the transduced CD8 T cells. While increased cross-reactivity by the mouse high-affinity TCR m33 in CD8 T cells resulted in deletion (74–76), several clinical trials in humans resulted in dangerous pathologies caused by the introduced T cells. The reasons for the difference in outcome are not entirely clear. One possibility is that the expression pattern of the cross-reactive epitope influences the outcome; for example, one cross-reactive epitope with the high-affinity m33 TCR, dEV8, is expressed ubiquitously, possibly overwhelming the introduced CD8 T cells and leading to AICD or even fratricide. By contrast, for cross-reactive epitopes that are tissue restricted (see below), the T cells may be able to persist and ultimately to mediate localized tissue destruction. Using appropriate animal models with tissue-restricted antigens, and adoptively transferred T cell with higher-affinity TCRs, it should be possible to investigate systematically the cause for different outcomes.

## Affinity of the TCR Correlates with Reactivity for Structurally Related Peptides

Given the central role of the TCR:pepMHC interaction in activity and specificity it is not surprising that significant efforts have gone into dissecting the interface, often residue by residue. Of particular relevance is the role that TCR affinity plays in the recognition of structurally similar peptides, as such peptides could represent potential off-target safety issues. In order to consider this issue, we provide below a non-exhaustive review of several systems: the mouse class I pepMHC-specific TCR (2C), a mouse class II pepMHC-specific TCR (3.L2), and human TCRs against the cancer antigens MART-1, NY-ESO, MAGE-A3, and WT1. We focus on the activities mediated against the cognate peptides and, where available, structurally related peptides.

### Mouse 2C TCR against class I antigens

The murine 2C T cell system (77, 78) has been studied extensively, from the level of central tolerance (79), to the level of structure/function (80–82), to its use in many tumor models (76, 83, 84). The CD8 T cell clone 2C was induced in a BALB.B mouse (H-2^b^) by an alloresponse to the H-2^d^ tumor P815 (85). The 2C TCR was shown to mediate positive-selection by K^b^ (79), and a potential self-peptide, called dEV8, involved in this selection has been identified (86, 87). A synthetic peptide, called SIY, that acts as a strong agonist in the context of K^b^ was also identified (88).

The known reactions of 2C with a variety of ligands (K^b^, L^d^, and K^bm3^) have provided a model system to study TCR degeneracy (89). Affinities for the allogeneic ligands [p2Ca/L^d^ and QL9/L^d^ *K*_d_ ∼ 1 μM (90, 91)], the putative positive-selection ligand [dEV8/K^b^, *K*_d_ ∼ 80 μM (90)], and the strong agonist ligand [SIY/K^b^, *K*_d_ ∼ 30 μM (26, 90, 91)] have been measured by various methods. The structure of this receptor in complex with dEV8/K^b^ was the first mouse TCR:pepMHC to be determined (80). Since then, the structures of the 2C TCR in complex with L^d^ ligands (81, 82) and K^b^ ligands (80, 92) have been solved, showing how the complementarity determining regions (CDR) accommodate the various ligands. CD8 2C T cells, have also been used to probe the exquisite sensitivity of T cells, suggesting that only a few agonist pepMHC molecules (or even one) on a target cell can mediate activity (33, 34). Finally, the 2C system and the strong agonist peptide SIY was used by Schreiber and colleagues to reveal the process of tumor antigen cross-presentation on stroma (83, 93, 94), and more recently the system has been exploited by Jacks and colleagues to reveal aspects of peripheral tumor tolerance (84) and the importance of mutated peptide antigens in immunoediting (95).

**Figure 2:**
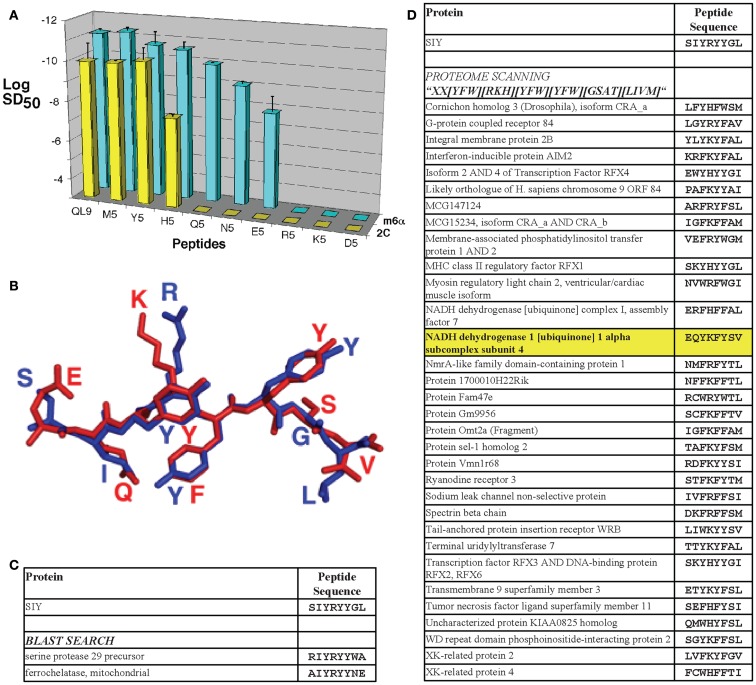
**T cell receptor affinity, specificity, and cross-reactivity in the 2C TCR system**. **(A)** The m6α TCR engineered from the 2C TCR for increased affinity for QL9/L^d^ exhibited more sensitive reactivity with structurally related peptides with single-amino acid substitutions. Sensitization doses of various QL9 position 5 variant peptides for IL-2 production by CD8-negative TCR transfectants are shown. The log of the SD_50_ value was plotted for each of the peptides used to stimulate 2C TCR (yellow bars) and m6α TCR (blue bars) transfectants [*Reproduced with permission from Ref. (56)]. **(B)** The 2C TCR reacts with the agonist SIY peptide/K^b^ complex and the putative positive-selecting peptide dEV8/K^b^ complex with *K*_d_ values of 30 and 80 μM, respectively. While the sequences share only two amino acids in common, they are structurally very similar [shown here aligned from their H2-K^b^-bound structures, SIY in blue and dEV8 in red; PDB IDs 1G6R (92) and 2CKB (173)]. **(C)** Performing a protein BLAST search of the mouse proteome with the SIY peptide sequence string and an Expect value cut off of 5.0 yielded only two sequence-similar peptides. **(D)** Performing a proteome scan to find sequences similar to SIY, based in part on alanine scan data of the peptide epitope, and the tolerance for mutations at each position yielded 43 peptides considered to be similar (only 33 sequences predicted to bind with SYFPEITHI scores>16 are shown). Using this technique, the putative positive-selecting peptide, and the self-peptide that reacts with the higher affinity TCR m33 (71), called dEV8 was identified (shown in bold, highlighted in yellow).

In the context of the present review, the 2C TCR (*K*_d_ = 1 μM for QL9/L^d^, and 30 μM for SIY/K^b^) was also the first to be engineered for higher affinity by directed evolution, first against QL9/L^d^ (96) and then against SIY/K^b^ (71). A yeast display library of CDR3α mutants in the 2C single-chain TCR (scTCR) were selected with QL9/L^d^ to yield various mutants, including m6 with a *K*_d_ value of 10 nM (91, 96, 97). The same 2C scTCR library, selected with SIY/K^b^, yielded various mutants including m33 with a *K*_d_ value of 30 nM (26, 71, 91). Stimulation of a T cell hybridoma expressing the higher-affinity TCR variants showed that they exhibited increased sensitivity to agonist peptide presentation (71, 97). In addition to sensitive agonist responses, binding of high-affinity TCR variants to structurally related pepMHC complexes were also increased (Figure 2A) (39, 71, 96, 97).

In addition to a broader range of reactivity with single-amino acid substitutions in the agonist peptide, the higher-affinity TCR m33 (isolated against the ligand SIY, with 1000-fold higher affinity) also showed CD8-dependent activity against the structurally similar self-peptide dEV8 (71). Although the m33 TCR only exhibited about a twofold increase in affinity for the self-pepMHC dEV8/K^b^, this increase was sufficient for CD8 T cells expressing m33 to be stimulated by both exogenous dEV8 and endogenous peptides presented by H-2^b^ cells such as C57BL/6 splenocytes (71). While the sequence of dEV8 only contains two amino acids in common with the strong agonist SIY (SIY: SIYRYYGL; dEV8: EQYKFYSV), they are very similar structurally (Figure 2B), and can be considered to be analogous to single-amino acid substitutions of agonist peptides. This notion forms the basis of the more detailed discussion below concerning the examination of structurally similar self-peptides.

It is important to point out that in contrast to an increase in affinity for structurally similar pepMHC complexes (i.e., m6 TCR with QL9 and its variants, or m33 TCR with SIY and dEV8), the affinities of engineered 2C variant TCRs were not increased toward structurally dissimilar ligands. For example, the high-affinity TCRs m6 and m13 selected against the allogeneic ligand QL9/L^d^, had reduced affinities for the syngeneic ligand SIY/K^b^ (91).

### Mouse TCR 3L2 and its ligands

Similar effects of increased affinity were observed for the class II-restricted TCR system called 3.L2 (98, 99). The 3.L2 TCR was derived from a CD4 T cell clone against a peptide from the minor d allele of the b chain of mouse hemoglobin, presented in complex with I-E^k^. The 3.L2 TCR was engineered by yeast surface display for increased affinity to the Hb/class II pepMHC complex. A panel of TCRs with an affinity range from the wild-type 3.L2 [*K*_d_ 20 μM (99, 100)] to the highest affinity variant, m15 (*K*_d_ 25 nM) were isolated (99). In the case of these higher-affinity TCR variants, there were no apparent increases in CD4 T cell activity for the agonist pepMHC. This may be a result of a wild-type affinity already above the optimal activation threshold for this complex. However, the ability to respond to single-amino acid substitutions of the Hb peptide was much broader for the TCRs with increased affinity (99, 101). A recent study showed that even a TCR (m2) with a modest improvement in affinity (twofold) for Hb/I-E^k^ mediated broader peptide reactivity, and enhanced thymic negative selection (102). Thus, like the 2C system, the 3.L2 system also showed that structurally similar peptides have a higher probability of stimulating T cells that express affinity-enhanced TCRs.

### Human TCRs

A prioritized list of cancer-associated peptide antigens has been compiled, setting quantitative values on various properties, including antigenicity, relationship to oncogenicity, and specificity (103). Among the panel of peptides, some have been the antigenic peptides targeted by TCRs in adoptive T cell therapies. These include, most prominently, MART-1 (29), NY-ESO-1 (104, 105), MAGE-A3 (106), and WT1 (107, 108). Various strategies to improve the affinity, and it is hoped thus the efficacy, of TCRs for the adoptive T cell therapy trials have been taken. While anti-tumor responses have been observed, there have been serious adverse events with MART-1 TCRs due to on-target/off-tumor activity (109), and lethal events with MAGE-3 TCRs due apparently to off-target cross-reactivity with structurally similar epitopes (110, 111). For these reasons, we summarize below various aspects of reactivities mediated by TCRs against four of the candidates for adoptive T cell therapies (MART-1, MAGE-A3, NY-ESO-1, and WT1).

#### MART-1

MART-1, a differentiation antigen upregulated on the surface of melanoma cells, contains the well-studied HLA-A2-restricted peptide epitope AAGIGILTV [27–35] (112) and its N-terminal extended variant EAAGIGILTV [26–35] (113). CD8 T cell clone M1F12 (now called DMF4) against this peptide was isolated from a patient with an anti-tumor response (114). The DMF4 TCR has a relatively low affinity (*K*_d_ 170 μM) for the predicted endogenous epitope, AAGIGILTV/HLA-A2 (115). The DMF4 TCR was used in one of the first trials of gene modified adoptive T cell transfer in humans (116). While relatively low overall response rates were reported [4/31, or 13%, with 17 patients reported in the original publication (109, 116)], the study represented an important step toward proof of concept for TCR gene therapies.

In an attempt to improve the efficacy of MART-1-directed TCR gene therapy, a second generation T cell clone called DMF5, with higher functional avidity and detectable activity in CD4 T cells, was isolated (117). The affinity of DMF5 (*K*_d_ 40 μM) (115) was higher than DMF4, but interestingly still lower than the murine wild-type receptor 2C (*K*_d_ 30 μM) [Note: like DMF5, the 2C TCR exhibited some activity in CD4 T cells *in vitro*, although *in vivo* anti-tumor activity of CD4 T cells with the 2C TCR was less effective than the higher-affinity TCR m33, with a *K*_d_ of 30 nM (76)]. Similarly, because DMF5 showed greater *in vitro* activity than DMF4 in CD4 T cells, it was hypothesized that DMF5-transduced T cells might mediate improved anti-melanoma responses (109). Indeed, objective response rates were higher in the DMF5 trial (30 vs. 13%). However, unlike patients treated with DMF4, patients treated with DMF5 experienced a marked cytokine (IFN-γ) spike and serious skin rashes 3–5 days after T cell transfer. The cytokine spike induced was ∼9-fold higher for patients treated with the affinity-enhanced DMF5 TCR when compared with previous patients who received cells with the DMF4 TCR, suggesting that the TCR reactivity was related to these results. Furthermore, since IFN-γ is produced by activated T cells, and patients were lymphodepleted prior to transduced T cell infusion (and still showed signs of lymphodepletion at the 3- to 5-day time point), it is likely that the cytokines were derived from the transferred cells. Importantly, DMF5 also mediated high rates of anterior uveitis, hearing loss, and dizziness, presumably due to reactions to MART-1 expressed in the normal eye and ear (109). Accordingly, these responses were characterized as on target/off tumor, and were only revealed by the potency of T cells transduced with the higher-affinity DMF5 TCR.

#### MAGE-A3

MAGE-A3 is a cancer-testis antigen and a member of a larger MAGE family. A related family member, MAGE-A1, was the first immunogenic gene found to elicit a natural CTL response in a melanoma patient (118). MAGE-A3 was identified several years later (119) and is one of the most commonly expressed MAGE family genes in cancers of different epithelial origins [reviewed in (120)]. Several peptide epitopes from MAGE-A3 have been identified, restricted by various MHC alleles. Here, we focus on the HLA-A2-restricted epitope, MAGE-A3 [112–120]: KVAELVHFL, which was the epitope targeted in a recent trial that resulted in the deaths of two patients (111), although a recent clinical trial with a MAGE-A3 epitope (EVDPIGHLY [161–169]) restricted by HLA-A1 also showed cross-reactivity, cardiovascular toxicity, and lethality in a clinical trial (110).

A high-avidity TCR was generated by vaccinating an HLA-A2 transgenic mouse with the MAGE-A3 [112–120] peptide (106). As murine CD8 does not bind efficiently to HLA-A2, T cells generated against peptide/HLA-A2 complexes in these mice presumably have affinities above the CD8 independence threshold, and would be sufficient to recruit CD4 as well as CD8 T cells. Human CD8, but not CD4, T cells expressing the MAGE-A3 [112–120]-specific TCR stained with soluble pepMHC tetramers, and were activated *in vitro* by MAGE-expressing tumor cells. To identify a TCR with even higher avidity, various point mutations in the CDR3α were examined for improved T cell activity (29), revealing an A118T variant that raised the functional avidity of the TCR, and mediated improved CD4 T cell activity (106). T cells transduced with these MAGE-A3/HLA-A2 TCRs were also screened against structurally similar peptides from other MAGE family members. An epitope from MAGE-A12 (differing only by a Val to Met substitution at position 2) was recognized indistinguishably from MAGE-A3, and detectable responses were seen with similar peptides from MAGE-A2 and MAGE-A6.

The MAGE-A3 A118T TCR was recently used in adoptive T cell therapy in nine melanoma patients (111). Five patients experienced objective regression of their tumors, including one complete response and one durable partial response that persisted for over 12 months. However, unexpected neurological toxicity was observed in three MAGE-A3 patients, resulting in two patient deaths. High levels of CD4 T cells with the murine TCR were detected in the cerebrospinal fluid of the patients that experienced toxicity, although brain infiltrating T cells were predominantly CD8 (CD4 T cells were rare). Cells expanded from the cerebrospinal fluid of one of the patients who succumbed showed specific IFN-γ release when stimulated with MAGE-A3^+^/HLA-A2^+^ tumor cells.

To identify potential cross-reactive epitopes that might have accounted for these toxicities, a BLAST search of the MAGE-A3 peptide, KVAELVHFL, was conducted with the human genome, revealing various candidates (111). The peptides were synthesized and tested for their ability to stimulate CD8 T cells transduced with the MAGE-A3 A118T TCR. One peptide (SAAELVHFL from EPS8L2, for epidermal growth factor receptor kinase substrate 8-like protein 2) was reactive, but transfection of the full EPS8L2 gene into HLA-A2-positive cells did not stimulate activity. However, staining of brain sections with anti-MAGE family antibodies, as well as testing with Q-RT-PCR, revealed a subset of neurons that expressed MAGE genes, including MAGE-A12 (111). Thus, it was suggested that T cell recognition of the structurally similar peptide from MAGE-A12 likely accounted for the neuronal toxicity.

#### NY-ESO-1

NY-ESO-1 (or LAGE-1) is also a cancer-testis antigen that is expressed on a variety of tumors from different origins [reviewed in (121)]. An NY-ESO-1 peptide (NY-ESO-1 [157–165], SLLMWITQC) restricted by HLA-A2 was identified using CTL lines from a melanoma patient (122). A CD8-dependent TCR called 1G4 that is specific for this epitope was shown to have *K*_d_ value of 15 μM for the NY-ESO/A2 complex (104, 105).

As the native NY-ESO peptide bound poorly to HLA-A2, and was less active in solution due to reactions of the C-terminal cysteine (123), there have been efforts to design improved peptide analogs. Toward this effort, a positional alanine scan (124) indicated that P3-Leu, P4-Met, P5-Trp, P7-Thr, and P8-Gln were important for T cell recognition, while a crystal structure of the HLA-A2-bound peptide (125) showed that P2-Leu, P3-Leu, P6-Ile, and P9-Cys were unlikely to contact the TCR directly. To eliminate the problems with the cysteine at P9, and to improve HLA-A2 binding, various P9 substitutions have been tested (104, 123, 125). A peptide with a valine substitution (SLLMWITQV) bound better to A2, was more stable in solution (123), and stimulated 1G4 T cells more effectively than the wild-type peptide *in vitro* (104). However, vaccination strategies with the C165V peptide did not lead to efficient cross-reactivity with the wild-type peptide (126), likely due to a repositioning of the peptide main chain with the different anchor residue at P9 (127).

While vaccination for NY-ESO-1 remains challenging, adoptive T cell therapy for this epitope has been shown to be effective and safe, even with a higher-affinity TCR variant of 1G4. Several single-site CDR mutants of the 1G4 TCR increased affinity and mediated improved activity of CD4 T cells (29). The 1G4-α95LY TCR has been tested clinically in melanoma and synovial cell sarcoma with a significant benefit (overall response rate of 45 and 67%, respectively), and a good safety profile has been reported for the 17 treated patients (128).

In a separate strategy, the 1G4 TCR has been modified for higher affinity by phage display yielding affinities as high as 26 pM (129, 130). The highest affinity TCRs yielded self-reactivity in both CD8 and CD4 T cells (73) (Figure 1). A high-affinity (50 pM) variant generated by phage display has also been produced as a soluble, bispecific fusion with anti-CD3 to redirect T cells to NY-ESO *in vitro* and in a human xenograft model in mice (131, 132).

#### WT1

Wilms’ tumor antigen (WT1) is a zinc-finger transcription factor that plays a significant role in embryogenesis but is minimally expressed in normal adult tissues. It is overexpressed in most leukemias, and in several other tumor types [reviewed in (133, 134)]. The recent prioritization of tumor-associated peptides (103) ranked WT1 as the top target due to its immunogenicity, restricted expression in normal tissues, and a strong correlation with tumorigenesis. An immunogenic HLA-A2-restricted epitope, WT1 [126–134]: RMFPNAPYL has been characterized (107, 135). Interestingly, the identical peptide sequence is present in the mouse WT1 homolog, and has been shown to be an immunogenic epitope in the context of H2-D^b^ (136, 137). [Note: a TCR targeting WT1 [235–243], restricted by HLA-A*2402 (138), is also being explored for adoptive immunotherapy, with reported efficacy and safety in pre-clinical systems (139); this peptide and TCR are not discussed further here.]

To date, several WT1 vaccination trials in mice and humans have been undertaken, showing excellent safety profiles but low response rates [reviewed in (140)]. A recent study (141) showed that only clones of low functional avidity for HLA-A2:WT1 [126–134] could be isolated from HLA-A2-positive individuals, while clones of higher functional avidity could be obtained from HLA-A2-negative individuals through allogeneic stimulation *in vitro*. However, the allogeneic clones showed promiscuous reactivity to different HLA-A2-bound peptides (141), highlighting that caution should be taken when taking advantage of allogeneic stimulation to isolate tumor-specific TCRs of improved affinity. A limited trial where anti-WT1 CTL clones were elicited *ex vivo* from patients, in the presence of IL-21, and re-introduced showed substantial persistence of the WT1-specific T cells (108). The results also suggested an improved response over WT1 vaccines, while maintaining favorable safety. Looking toward adoptive T cell therapy, a WT1 [126–134]/A2-specific TCR isolated from peptide-specific, allo-induced CTLs (107, 142), exhibited good anti-tumor responses in a mouse xenograft model with TCR-transduced T cells (143, 144). A more recent study targeting WT1 for adoptive T cell therapies described a novel strategy to reduce endogenous TCR levels by using a targeted zinc-finger nuclease, followed by introduction of their WT1-specific TCR. This approach resulted in enhancement of overall functional avidity due to the higher T cell surface levels of the exogenous WT1-specific TCR (145).

With the possibility for improvement of anti-WT1 CD4 T cell responses with higher-affinity TCRs, our lab, working with Greenberg and colleagues has previously engineered an enhanced affinity (CD8-independent) TCR against the murine WT1/D^b^ complex (137, 146), and we have recently engineered a higher-affinity human TCR against WT1/HLA-A2 (unpublished). The mouse and human TCRs are being tested in mouse models with analysis of potential on-target/off-tumor responses, or cross-reactivity with structurally related pepMHCs (see below). Adoptive transfer studies with CD8 T cells and the mouse TCR against WT1/D^b^ have shown no signs of toxicity in the mouse models (146).

## Does the Advantage of Higher-Affinity TCRs in CD4 T Cells Outweigh the Potential Disadvantage with Self-Reactivity?

Given the connection between sensitivity and cross-reactivity with TCRs in CD8 T cells, it is reasonable to ask if the recruitment of CD4 T cells with higher-affinity TCRs is worth the risk of self-reactivity (by transducing all peripheral T cells including CD8 T cells). As described above, redirected CD4 T cells provide an opportunity for direct destruction of the tumor by the effector CD4 T cells. Our recent findings (76) and results from others (75) suggest that nanomolar affinity TCRs are more potent in CD4 T cells than wild-type TCRs. In fact, the only treatment which resulted in long-term control of established tumors, with no outgrowth, was CD4 T cells transduced with the 30-nM affinity TCR m33 (76).

We suggest that the major importance of CD4 T cell recruitment will be that they provide a cytokine milieu that facilitates the generation of endogenous responses against multiple class I MHC-restricted cancer antigens. These antigens might include individual unique peptides with tumor-specific, patient-specific mutations. Such mutated peptides have recently been shown to represent the dominant epitopes of an effective immune response that drives immunoediting (95, 147). Accordingly, it will be important to identify strategies that allow TCRs to mediate CD4 T cell activity, ultimately enabling a broad anti-cancer immune response. Since adoptive T cell therapies currently are configured to introduce the same TCR into both CD4 and CD8, an important issue is whether it is possible to improve current pre-clinical approaches to assess potential self-reactivity and consequent toxicity.

One possible strategy to take advantage of high-affinity TCRs in immunotherapy would be to separate CD4 and CD8 T cells *ex vivo* for transduction with separate TCR variants, as has been done in mouse studies (74–76). The CD4 T cells could be transduced with nanomolar affinity TCRs, while the CD8 T cells could be transduced with a reduced affinity version of the receptor. The method for creating a lowered affinity version of a TCR is fairly straightforward, as conserved residues in the CDR2 loops may be substituted, reducing the overall binding affinity of the TCR while maintaining the peptide specificity. Using a library of receptors with different residues at a single CDR2β position in the m33 and 2C TCRs, we recently showed that a range of binding affinities were achieved by the resulting receptor variants, and certain variants were sufficiently lowered in affinity to minimize cross-reactivity in CD8 T cells, but retain CD4 T cell activity (74). Several conserved positions in TCRs have been characterized [reviewed in (148)] which could be mutated to achieve lower-affinity variants of an anti-tumor TCR.

It is of course possible that adoptive T cell therapy could be combined with checkpoint blockade treatment to interfere with negative signals transmitted to T cells, for example from interactions of molecules such as CTLA-4 or PD-1 and their ligands, B7 and PD-L1 or PD-L2, respectively [reviewed in (149)]. With the advent of checkpoint blockade treatments, including antibodies that inhibit CTLA-4 (FDA-approved Ipilimumab) and PD-1 (or its ligand PD-L1), it is possible that lower-affinity TCRs will have improved efficacy in adoptive T cell therapies. Clinically, checkpoint blockade [reviewed in (150)] has been used to enhance endogenous T cell responses against a tumor. In melanoma patients, ipilimumab treatment showed a survival benefit, either alone or with a gp100 peptide-based vaccine, over the peptide vaccine alone (151). Patients treated with ipilimumab often exhibited tissue restricted, immune-related adverse autoimmune effects. Recently, there has been considerable excitement about blocking PD-1 signaling. As the PD-1 ligands, PD-L1 and PD-L2, are specifically upregulated at sites of inflammation and on many tumors (149), PD-1 blockade may more directly target immunosuppression in the tumor with fewer side effects than with CTLA-4. PD-1 blockade, currently in clinical trials in the form of several different antibodies (152–155), has shown promising response rates [up to 52% objective response rate in advanced melanoma patients treated with the MK-3475 (lambrolizumab) PD-1 blocking antibody (155)], but these treatments were also associated with immune-related adverse effects, although at lower rates than CTLA-4 blockade. In combination with checkpoint blockade, it is possible that a lower-affinity TCR could act with higher potency in an adoptive T cell therapy setting, as has been seen in a mouse model (156). It remains to be seen if this may have similar safety concerns as with higher-affinity TCRs, in terms of cross-reactivity, or on-target/off-tumor responses.

## Potential Strategies to Evaluate Self-Reactivity Risks Associated with Higher-Affinity TCRs

Along with the promise of adoptive therapy with engineered TCR-transduced T cells has come the very real dangers of on-target/off-tumor toxicity (as seen in the MART-1 trial) and cross-reactivity with similar epitopes in normal tissues (as seen in the MAGE-A3 trial). Several important techniques are already in use to check for cross-reactivity, including *in vitro* screening of CD4 and CD8 T cells transduced with tumor-specific TCRs, using as antigen-presenting cells various lines derived from normal tissues. However, to safely take advantage of this therapeutic strategy and avoid serious adverse effects, it will be imperative to develop expanded strategies to screen candidate TCRs for safety and potential cross-reactivity prior to delivery into human patients. We propose below a combination of *in silico*, *in vitro*, and *in vivo* (murine) strategies to enhance current screens prior to clinical trials. In each case, we argue that having an engineered, high-affinity TCR would be of significant value in revealing potential safety concerns, even if a lower-affinity TCR may be desirable in a clinical setting, especially in CD8 T cells (75, 76). It is relatively easy to introduce mutations at one of several, well-characterized locations in the TCR [reviewed in (148)] that can reliably reduce affinity while maintaining specificity and anti-tumor activity (74).

One way to attempt to detect possible cross reactivities for a given TCR will be to take advantage of the vast amount of information available through genomic and proteomic databases. A standard protein BLAST (Basic Logical Alignment Search Tool, blastp algorithm)^1^ search can be conveniently performed using the NCBI web interface, revealing similar sequences to a given peptide ranked with an Expect (E) value. The E value is a measure of the statistical significance of a particular match compared to random chance in the entire proteome, with lower E values being more significantly similar to the search string. As a model, the mouse proteome was searched by BLAST for sequences similar to the SIY peptide, which acts as an H2-K^b^-restricted agonist for the 2C TCR, but is not actually contained within the mouse proteome. This search revealed two peptides with Expect values<5.0 (Figure 2C). However, the previously identified positive-selecting antigen, dEV8, was not identified in the BLAST search, even extending the accepted E value up to 10,000. Thus, BLAST searches alone do not capture the criteria that would be best used to search for MHC-binding peptides with potentially similar TCR-contact residues.

**Figure 3:**
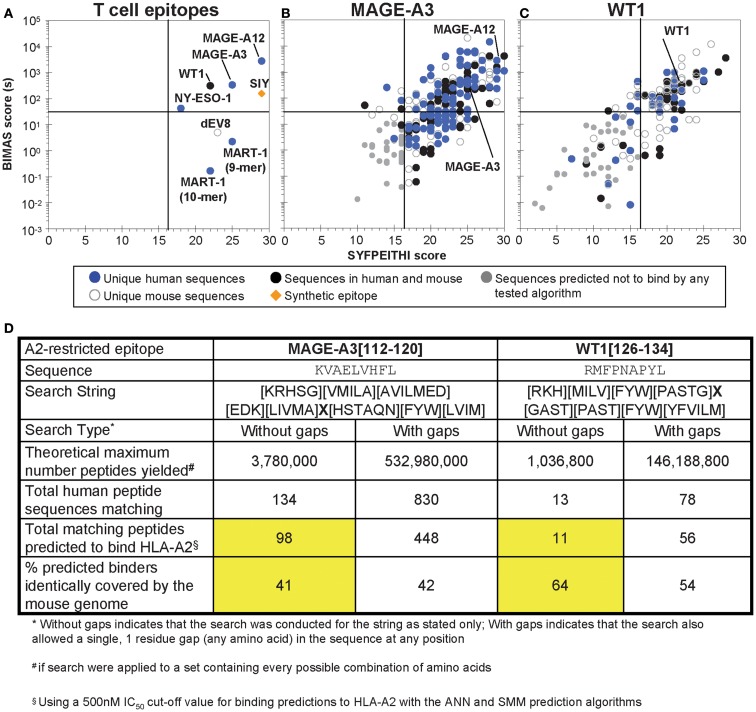
**Analysis of selected tumor epitopes for homologous sequences in the human and mouse proteomes**. **(A)** MHC-binding prediction scores for a set of characterized T cell epitopes, including six HLA-A2-restricted human tumor-associated epitopes. **(B)** MHC-binding predictions for peptides identified in a MAGE-A3 [112–120] homology scan (no gaps) of the human and mouse proteomes. **(C)** MHC-binding predictions for peptides identified in a WT1 [126–134] homology scan of the human and mouse proteomes (including an allowed, single-amino acid gap). For **(B,C)**, peptides were subjected to ANN and SMM prediction algorithms along with SYFPEITHI and BIMAS, and prediction to bind above the arbitrary thresholds described in the text in any of the algorithms was taken to indicate a potential binder. **(D)** A comparison of MAGE-A3 and WT1 proteome scan results. The total number of predicted binders identified in the human proteome and the percent of the binders identically found in the mouse proteome for both epitopes (searched without gaps) are highlighted in yellow.

All peptides identified through *in silico* screens were tested in MHC-binding prediction algorithms with arbitrary cut-off values used previously for distinguishing qualitative binders vs. non-binders. Algorithms examined here included SYFPEITHI with a cutoff of>16 for binders (157)^2^; BIMAS with a cutoff of estimated *t*^½^ > 30 s^3^; Artificial Neural Network [ANN; (158)], and Stabilized Matrix Method [SMM (159)]. ANN and SMM were both applied with a cut-off value of IC_50_ < 500 nM, and both were accessed through the Immune Epitope Database (IEDB) Analysis Resource^4^. A plot of predicted MHC-binding values for epitopes discussed in this review are shown in Figure 3A. It has been estimated that an IC_50_ cutoff of 500 nM by ANN or SMM yields 80% or higher (up to 97%) accuracy in predicting MHC binders, depending on allele (160). However, it is important to keep in mind that some MHC-binding peptides may be missed using a threshold such as this; for example, using 500 nM as a binding threshold for netMHCpan H2-K^b^ binding predictions would omit p2Ca, a peptide which is known to form a complex with H2-K^b^ and stimulate 2C T cells (161).

Using the MHC-binding principles, an alternative strategy to BLAST is to scan the full proteome for sequence motifs that: (1) preserve critical residues (or conservative mutations) ideally identified by positional single-site substitutions of the peptide epitope, and (2) allow other residues to vary more widely. A similar strategy was used to identify potential positive-selecting ligands for the OT-1 TCR, scanning for MHC-binding motifs, and then scoring for similarity among the predicted TCR contacts to the H2-K^b^-restricted ovalbumin peptide, OVA (87). For our current efforts, using previous alanine scan information for the SIY peptide (SIYRYYGL) that stimulates the 2C TCR (39), a search motif was designed as “*XX[YFW][RKH][YFW][YFW][GSAT][LIVM]*,” where “X” indicates that any residue would be acceptable at that position, and bracketed residues indicate that one of those limited set of residues would be acceptable. If every possible sequence were available in the proteome, the 2C homology search, as designed, would yield 518,400 sequences (20 × 20 × 3 × 3 × 3 × 3 × 4 × 4). Scanning the mouse proteome [the complete *Mus musculus* proteome from The Universal Protein Knowledgebase (UniProtKB), 73,947 entries] with this motif identified 43 peptides, of which 33 were predicted to bind well to H2-K^b^ (in this case, defining binders using a SYFPEITHI cutoff of 17 or higher, see Figure 2D). Importantly, this strategy identified, among the 33 peptides, dEV8 from the NADH dehydrogenase, which as described above is known to react with 2C TCR and the higher-affinity m33 TCR.

We propose that the yield of identified, predicted MHC-binding peptides when searching the proteome in this manner provides a reasonable estimate of the possible number of self-reactive peptides, and a tractable number of candidates that could be tested for reactivity with a higher-affinity TCR (like m33) in CD8 T cells. Of course, the identified peptides are influenced by the design of the search string, as well as the accuracy of binding predictions. To improve the ability of searches like this to comprehensively identify all potentially cross-reactive peptides, an epitope of interest should be evaluated for the ability of substituted peptides to activate its specific TCR. Value can be obtained from simple, single-point alanine substitutions, as can be seen from the ability of the murine proteome scan to identify the dEV8 peptide (with only two amino acids in common with the agonist peptide, Figure 2D) when guided by alanine substitutions of SIY for binding and stimulation of the 2C TCR (39).

While the search strategies for structurally similar peptides may identify potential problematic cross-reactive epitopes, this strategy alone can not identify structurally dissimilar peptides which act as agonists. It is possible that such peptides could be identified using combinatorial peptide library techniques, where individual positions/residues are held constant in each peptide pool, and stimulation is evaluated (162, 163).

To evaluate *in silico* strategies for human tumor targeting, BLAST searches of the HLA-A2-restricted epitopes for MART-1, NY-ESO-1, MAGE-A3, and WT1 were performed in the human proteome; similarity was defined with an Expect value cut off of 5.0. Within this range of similarity, the MART-1 (27–35) and WT1 (126–134) described above were identified as unique within the proteome. NY-ESO-1 (157–165) yielded two additional peptides rated similar within these criteria; however, neither was predicted to bind to HLA-A2. By contrast, MAGE-A3 (112–120) yielded fourteen sequences that were similar within this range of Expect values, of which 10 were other members of the MAGE family. All of the MAGE-similar peptides were predicted to have some binding to HLA-A2 (SYFPEITHI score greater than 16 or BIMAS off-rate>30 s).

Two of the epitopes, MAGE-A3 and WT1, were used further as the basis for a scan for structurally similar peptide sequences, as done with SIY. The contribution of each peptide position to T cell recognition has not been systematically studied for these epitopes; however, some data on substitutions is available (106, 111, 164–166). Using these data, and striving to maintain structural homology/conservative mutations, proteome search strings were generated, and applied to both the human and murine proteomes. This strategy thus further aimed to determine what fraction of potentially cross-reactive, structurally similar epitopes would be represented in both the human and murine proteomes. This information could be useful in examining what fraction of potential cross-reactive epitopes might reveal toxicities in a mouse model (see below).

If every possible sequence were available in the proteome, the MAGE-A3, and WT1 searches would yield ∼3,800,000 and ∼1,040,000 sequences, respectively. Applying the searches to the human proteome (the complete *Homo sapiens* proteome downloaded from UniProtKB, 134,787 entries) yielded 134 sequences similar to the MAGE-A3 epitope and 13 sequences similar to the WT1 peptide. Thus, consistent with the BLAST search, WT1-like sequences were about 10-fold more rare in the human proteome than MAGE-A3-like sequences. We also allowed the search string to include a single random amino acid gap anywhere in the peptide sequence for WT1, yielding larger theoretical search maxima (e.g., 146,000,000 for WT1, almost 40-fold larger than the theoretical search size without gaps for MAGE-A3, 3,800,000). When even that search string for WT1 was applied to the human proteome, only 78 peptides were identified, still half as many as identified with the MAGE-A3 search string without gaps (134 peptides). It should be noted that for most TCRs, insertion of a single residue (i.e., “gap”) in the peptide may significantly alter the bound conformation of the peptide, resulting in a loss of recognition of the epitope. Using a combinatorial library scanning approach where peptide pools of different length were tested for the ability to stimulate different TCRs, it has been shown that TCRs have restricted length preferences in the peptide epitopes that they recognize (163). Thus, it remains to be seen whether the addition of “gaps” in a search string are of any value. This can be readily determined by activity analysis of cognate peptides that have the various single-amino acid insertions.

The MAGE-A3 and WT1-related peptides were further screened using the binding prediction algorithms listed above, and peptides predicted to bind by the ANN or SMM algorithms (IC_50_ < 500 nM) were designated as potential binders, resulting in 98 and 11 peptides for MAGE-A3 and WT1, respectively. Interestingly, of these epitopes, 41 and 64%, respectively, were identically represented in the mouse proteome, with many others having highly homologous sequences. The distribution of homologous sequences identified through these screens, and their presence uniquely in the human proteome, the mouse proteome, or identically in both is shown in Figures 3B,C (where the WT1-like peptides in Figure 3C also include those with single-amino acid gaps). A summary of the search results for these two epitopes can be seen in Figure 3D. The number of peptides identified by this type of search in all three cases (SIY, MAGE-A3, and WT1) is readily amenable to small-scale synthesis and *in vitro* testing for T cell stimulation by peptide-loaded, HLA-A2-positive APCs. We propose this straightforward screen to evaluate cross reactivities with structurally similar epitopes. Peptides with reactivities would be followed with more detailed analysis of gene transcript levels in different tissues, and studies of the ability of the gene-product to be processed and presented.

Proteome searches using a particular motif can not assess all potential cross-reactive peptide epitopes, especially those without structural similarity. Hence, we propose that an additional *in vitro* screening strategy may be useful. For example, an open reading frame (ORF) library (167, 168) covering genes from the human proteome would be transfected into HLA-A2-positive APCs. ORF libraries have been used in yeast two-hybrid systems toward mapping the protein “interactome,” (169) and recently the human ORFome is being developed in a lentiviral vector system, which would allow for convenient application to mammalian cell transduction (168, 170). This would provide another opportunity to identify unpredicted cross reactivities, and such libraries would provide a resource available for screening virtually any TCR, restricted by the appropriate HLA alleles.

Finally, we propose expanded use of HLA transgenic mice to screen for safety. As mentioned above, 40–65% of peptides identified in MAGE-A3 and WT1 homology screens were identical in mouse and human, providing a rationale for using a mouse screen to identify at least some of the potentially adverse cross reactivities. The system would ideally involve the use of mouse T cells transduced with human TCRs (human V regions linked to mouse C regions), as these would provide syngeneic cell:cell adhesion systems for optimal activities. TCR-transduced mouse CD4 and CD8 T cells could be transferred to the transgenic HLA-A2/D^d^ hybrid MHCs (AAD, available from Jackson Labs) which allows cells to present HLA-A2 peptide epitopes while still engaging mouse CD8. This system could be tested with various affinity TCRs in order to push the limits of safety and efficacy.

A significant advantage of the mouse system would be the opportunity to also generate additional transgenic mice on the AAD background, where the tumor gene of interest (e.g., MART-1, MAGE-A3, or NY-ESO-1) is expressed under the relevant mouse promoter. Such models could reveal on-target/off-tumor activities due to uncharacterized expression of the target gene in normal tissue, either at low levels or by a low-frequency cell subset. As the WT1 [126–134] epitope is identical in the mouse and human proteins, this provides an opportunity to assess safety without the generation of the human WT1 transgene.

## Concluding Remarks

While there will always remain a risk of unpredicted reactivities in patients receiving adoptive T cell therapies, we believe that the use of TCRs with different affinities and specificities in an expanded set of pre-clinical approaches, as described here, will identify some of the possible problems. Proteome search approaches provide a measure of the number of related self-peptides that could pose safety concerns with adoptive T cell therapies. In addition, the number of peptides represented in the proteome predicted to be similar to a given epitope should correlate with the extent of central tolerance that might exist against a cancer peptide. In this regard, this type of analysis might be considered for peptide vaccines (e.g., lower numbers of homologous peptides may correlate with higher frequencies of peripheral T cells that have escaped negative selection). Further safeguards at the initial clinical stage, such as reduction in the number of T cells delivered, may be considered. Significant progress has also been made in the development of suicide genes or alternative approaches that could allow rapid deletion of T cells before a dangerous reaction reaches the critical stage (171, 172). Finally, transfer of only CD4 T cells may be desirable as they can mediate strong anti-tumor effects and potential for helping endogenous immune responses, but CD4 T cells may not exhibit the CD8-dependent cross reactivities that the same TCRs mediate in CD8 T cells.

## Conflict of Interest Statement

The authors declare that the research was conducted in the absence of any commercial or financial relationships that could be construed as a potential conflict of interest.

## Footnotes

^1^http://blast.ncbi.nlm.nih.gov/

^2^http://www.syfpeithi.de/Scripts/MHCServer.dll/EpitopePrediction.htm

^3^http://bimas.dcrt.nih.gov/molbio/hla_bind

^4^http://tools.iedb.org/analyze/html/mhc_binding.html
